# [^177^Lu]Lu-DOTATATE for Recurrent Meningioma (LUMEN-1, EORTC-2334-BTG): Study Protocol for a Randomized Phase II Trial

**DOI:** 10.2967/jnumed.125.269633

**Published:** 2026-01

**Authors:** Nathalie L. Albert, Emeline Tabouret, Emilie Le Rhun, Felix Sahm, Julia Furtner, Joerg-Christian Tonn, Christian Alfano, José Pais Silva, Anne-Sophie Govaerts, Thierry Gorlia, Osvaldo Mirante, Giuseppe Minniti, Michael Weller, Matthias Preusser

**Affiliations:** 1 Department of Nuclear Medicine, LMU Hospital, Ludwig Maximilians University, Munich, Germany;; 2 APHM, Aix-Marseille University, CNRS, INP, Marseille, France;; 3 Service de Neurooncologie, CHU Timone, Marseille, France;; 4 Department of Medical Oncology and Hematology, University Hospital and University of Zurich, Zurich, Switzerland;; 5 Department of Neuropathology, University Hospital Heidelberg and Clinical Cooperation Unit Neuropathology, DKFZ and DKTK, Heidelberg, Germany;; 6 Research Center for Medical Image Analysis and Artificial Intelligence, Faculty of Medicine and Dentistry, Danube Private University, Krems, Austria;; 7 Department of Neurosurgery, LMU Hospital, Ludwig Maximilians University, Munich, Germany;; 8 EORTC Headquarters Team, Brussels, Belgium;; 9 Advanced Accelerator Applications, Geneva, Switzerland;; 10 Department of Radiologic Sciences, Oncology and Anatomical Pathology, Policlinico Umberto I, Sapienza University, Rome, Italy;; 11 Department of Neurology, University Hospital and University of Zurich, Zurich, Switzerland; and; 12 Division of Oncology, Department of Medicine I, Medical University of Vienna, Austria

**Keywords:** meningioma, [^177^Lu]Lu-DOTATATE, radiopharmaceutical therapy, theranostic, clinical trial

## Abstract

There are no established treatment options for patients with meningioma recurring after surgery and radiotherapy. Somatostatin receptor type 2 (SSTR2) is highly expressed in meningiomas, and SSTR2-targeting radionuclide therapy with [^177^Lu]Lu-DOTATATE has shown potential activity in the treatment of meningioma in uncontrolled and small studies. **Methods:** EORTC-2334-BTG (LUMEN-1, NCT06326190) is a randomized, multicenter, phase II trial in patients with recurrent World Health Organization (WHO) grade 1, 2, or 3 meningioma. In total, 136 patients will be randomized in a 2:1 ratio to [^177^Lu]Lu-DOTATATE (≤4 doses of 7.4 GBq given every 4 wk) or local standard of care (hydroxyurea, bevacizumab, sunitinib, octreotide, everolimus, or observation). The main eligibility criteria include age 18 y or older; neuropathologically confirmed meningioma of WHO grade 1, 2, or 3; WHO performance score of 0–2; measurable disease on MRI (≥10 × 10 mm); radiologically documented progression of any existing tumor (growth > 25% or new lesions) or appearance of new lesions within the last 2 y; SSTR positivity by PET imaging (SUV_max_ > 2.3); at least 1 prior surgery and at least 1 line of radiotherapy; and no prior systemic therapy. The primary efficacy endpoint is locally assessed progression-free survival according to Response Assessment in Neuro-Oncology MRI meningioma criteria, and secondary endpoints include radiologic response rate, overall survival, safety, health-related quality of life, and neurologic function. The trial protocol includes a comprehensive exploratory translational research program with dosimetry and imaging-based and tissue-based investigations. LUMEN-1 was activated in March 2025 and will enroll patients in 35 sites in 10 countries across Europe, with primary endpoint collection planned after 2 y and study completion after 5 y. To our knowledge, EORTC-2334-BTG (LUMEN-1, NCT06326190) is the first prospective randomized trial investigating the efficacy of [^177^Lu]Lu-DOTATATE in patients with recurrent meningioma.

Meningioma is the most common primary intracranial tumor in adults, with an average annual age-adjusted incidence rate of 10.63 cases per 100,000 individuals (1). According to the latest edition of the World Health Organization (WHO) classification of central nervous system (CNS) tumors published in 2021, meningiomas are categorized into 3 grades: CNS WHO grade 1 represents 75%–80% of all meningioma cases, CNS WHO grade 2 represents 20%–25%, and CNS WHO grade 3 represents 1%–6% (2). The prognosis of meningiomas differs across grades, where CNS WHO grade 1 meningiomas have recurrence rates of 7%–25%, whereas CNS WHO grade 2 meningiomas recur in 29%–52% of patients and CNS WHO grade 3 meningiomas recur in 50%–94%.

Maximal safe resection of meningioma and adjacent dura remains the primary therapeutic approach with curative intent (3). Radiotherapy or stereotactic radiosurgery is recommended for cases in which total gross resection is not achieved or for higher-grade tumors. The ongoing ROAM/EORTC-1308 trial is investigating whether early adjuvant external bean radiotherapy reduces the risk of tumor recurrence after complete surgical resection of atypical meningioma (4). At recurrence, there are no established treatment standards, and reresection, reirradiation, systemic pharmacotherapy, observation, and investigational treatments can be considered according to contemporary guidelines (3). Novel treatments for meningiomas recurring after local therapies (resection and radiotherapy) are strongly needed (5).

Radiopharmaceutical therapy is an emerging treatment approach in oncology. Radiopharmaceuticals composed of a carrier molecule with affinity to a specific target molecule are coupled via a linker to a radioisotope with the goal of delivering radiation doses directly to tumor cells or relevant components of the tumor microenvironment. Depending on the choice of radionuclide, diagnostic PET imaging and delivery of therapeutic radiation doses can be achieved, thus enabling selection of patients and tumors with an increased chance of response to targeted therapy (theranostic precision medicine).

[^177^Lu]Lu-DOTATATE (Lutathera; Novartis) is a radiopharmaceutical with a high affinity for somatostatin receptor type 2 (SSTR2). [^177^Lu]Lu-DOTATATE is approved by the European Medicines Agency and the U.S. Food and Drug Administration for the treatment of SSTR2-positive gastroenteropancreatic neuroendocrine tumors based on the results of the phase III NETTER trial (6,7). SSTR2 is overexpressed in most meningiomas (80%–95% of cases) and is considered a potential target for diagnosis and therapy (8,9). [^177^Lu]Lu-DOTATATE has been investigated in several small and uncontrolled studies of patients with meningioma (10,11). A recent individual patient data metaanalysis identified 6 eligible cohort studies comprising 111 patients with refractory meningioma treated with SSTR-targeting radiopharmaceutical therapy (12). Disease control was achieved in 63% of patients. The 12-mo overall survival (OS) rates were 88%, 71%, and 52% for WHO grades 1, 2, and 3, respectively. Furthermore, a recent study of 42 patients with progressive meningiomas reported a disease control rate of 57%, median progression-free survival (PFS) of 16 mo, and median OS of 36 mo (13). Recently, the interim results of an ongoing clinical trial (NCT03971461) that is evaluating the effect of [^177^Lu]Lu-DOTATATE in progressive intracranial meningiomas was reported (14). The trial is a single-arm, open-label, multicenter, phase II study performed in the United States with an overall sample size of 32 patients, following a Simon 2-stage design with PFS at 6 mo as the primary endpoint. At the interim report, 14 patients (11 women and 3 men) with progressive meningiomas (3 WHO grade 1, 10 WHO grade 2, and 1 WHO grade 3) have been enrolled. All patients previously underwent tumor resection and at least 1 course of radiation. Treatment with [^177^Lu]Lu-DOTATATE was well tolerated, and no treatment-limiting toxicities were observed. Of the 14 patients, 7 (50%) achieved PFS at 6 mo, and all 7 patients had achieved stable disease per radiographic evaluation by modified Macdonald criteria. A more than 25% reduction in [^68^Ga]Ga-DOTATATE PET was observed in 5 meningiomas and 2 patients. In 1 lesion, this corresponded to a more than 50% reduction in bidirectional tumor measurements on MRI. Although the data from the available small and uncontrolled studies indicate a potential therapeutic value of [^177^Lu]Lu-DOTATATE in meningioma, adequate evidence from randomized prospective clinical trials in this setting is lacking so far. Thus, SSTR2 is considered a hypothetical treatment target in meningiomas, with a score of IIIA on the European Society for Medical Oncology Scale for Clinical Actionability of Molecular Targets (15,16).

To our knowledge, EORTC-2334-BTG (LUMEN-1, NCT06326190), sponsored by the European Organisation for Research and Treatment of Cancer (EORTC), is the first prospective randomized trial investigating [^177^Lu]Lu-DOTATATE in recurrent meningioma. It will provide a head-to-head comparison of the efficacy of [^177^Lu]Lu-DOTATATE and local standard of care in recurrent meningiomas. Here, we summarize the clinical trial design of this ongoing study, with first site activation achieved in March 2025, primary endpoint collection planned after 2 y, and study completion after 5 y. The reporting guidelines of Standard Protocol Items: Recommendations for Interventional Trials for publication of clinical trials protocols (17) were used and are available in the supplemental materials (supplemental materials are available at http://jnm.snmjournals.org). The clinical trial is registered at ClinicalTrials.gov with the identification number NCT06326190. It was registered March 20, 2024, before the start of inclusion.

## STUDY DESIGN

**FIGURE 1.**
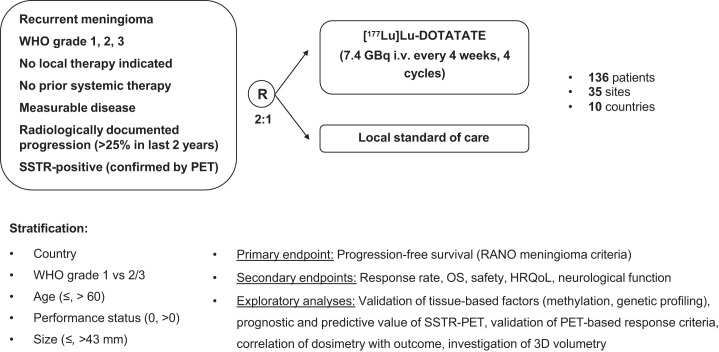
Study scheme of EORTC-2334-BTG (LUMEN-1) trial. i.v. = intravenously; RANO = Response Assessment in Neuro-Oncology; HRQoL = health-related QoL; 3D = 3-dimensional.

EORTC-2334-BTG (LUMEN-1) is a randomized, open-label, multicenter, phase II trial in patients with recurrent meningioma. Figure 1 summarizes the study design. The information provided reflects the protocol version 1.0 dated April 29, 2024. All necessary ethical and regulatory approvals by competent authorities are obtained before site activation. The study is submitted by the principal investigator, national coordinator, or sponsor, in accordance with local regulations, for review and approval by an appropriate independent ethical review committee or institutional review board and a national competent authority if required by the national laws of the countries where the study is conducted. All necessary approvals are collected by all participating sites before starting patient enrollment. All participating patients will provide written informed consent. Informed consent will be collected by the local investigators, who are board-certified physicians with current good clinical practice certification and with confirmed delegation and completed training for clinical trial activities.

Investigators must refer to the latest version of the full study protocol, which is available to study investigators in their trial files. Publicly available information and updates on the trial’s progress can be found at https://www.eortc.org/research_field/clinical-detail/2334/.

### Trial Population

The main eligibility criteria include age of at least 18 y; histologically confirmed diagnosis of meningioma (all grades, grades 1–3 per WHO, are eligible); WHO performance status of 0–2; measurable disease (≥10 × 10 mm contrast-enhancing lesion) on cranial MRI no more than 2 wk before randomization; radiologically documented progression of any existing tumor (growth > 25% in the last 2 y) or appearance of new lesions (including intra- and extracranial manifestations); SSTR-positive meningioma confirmed by PET imaging (with a locally established SSTR PET tracer) performed within 4 wk before randomization (baseline SSTR PET is considered positive when meningioma uptake intensity exceeds an SUV_max_ of 2.3); at least 1 prior surgery and 1 line of external beam radiotherapy (not further specified in the clinical trial protocol) for meningioma; adequate liver, renal, and hematologic function within 4 wk before randomization; no corticosteroid treatment or a dexamethasone dose of no more than 4 mg/d (or another corticosteroids’ equivalent dose) for a minimum of 7 d as initiation of the study treatment; no prior systemic treatment; and no local treatment (surgery or radiotherapy) indicated per the local investigator. Patients for whom local investigators consider external beam radiotherapy to be the best option are not eligible for LUMEN-1.

The detailed eligibility criteria are shown in Supplemental Table 1. The patients will receive extensive information about the study setup and requirements during recruitment by local investigators, who are board-certified physicians with current good clinical practice certification and with confirmed delegation and completed training for clinical trial activities. Clinical trial insurance according to the laws of the countries where the study will be conducted is in place for all participants.

### Objectives and Endpoints

The objectives and endpoints of the LUMEN-1 trial are detailed in Table 1. In brief, the primary efficacy endpoint is locally assessed PFS, and secondary endpoints include radiologic response rate, OS, safety, health-related quality of life (QoL), and neurologic function.

**TABLE 1.** Primary and Secondary Endpoints of EORTC-2334-BTG, Also Known as LUMEN-1, Trial

Endpoint	Description
Primary	PFS computed on basis of MRI-based RANO meningioma response criteria as assessed by local investigator
Secondary	BOR, defined as CR, MR, or PR during study treatment; objective CR, MR, or PR rate and median CR, MR, or PR duration, with CR rate and median CR duration computed by MRI on basis of RANO meningioma response criteria as assessed by local investigator
	OS, OS probability at 6 and 12 mo, and median OS
	Safety, CTCAE version 5.0, and tolerability, [^177^Lu]Lu-DOTATATE only
	Change from baseline in HRQoL in terms of global QoL, cognitive functioning, social functioning, and fatigue at week 24
	Change of neurologic function on NANO scale from baseline during study treatment

RANO = Response Assessment in Neuro-Oncology; BOR = best overall response; CR = complete response; MR = minor response; PR = partial response; CTCAE = Common Terminology Criteria for Adverse Events; HRQoL = health-related QoL; NANO = Neurologic Assessment in Neuro-Oncology.

PFS is defined as the number of days from the date of enrollment to the date of earliest disease progression based on response assessment or to the date of death due to any cause, if disease progression did not occur. Patients for whom neither death nor progression have been documented will be censored at the date of the last radiologic assessment at which the patient was progression-free.

OS is defined as the number of days from the date of enrollment to the date of death due to any cause. Patients known to be alive or dead after the analysis cutoff date are censored at the analysis cutoff date.

Radiologic follow-up for the evaluation of PFS and response rate will be performed by cranial MRI every 6 wk from enrollment for the first 9 mo and every 12 wk thereafter and as clinically indicated. MRI will be assessed according to Response Assessment in Neuro-Oncology meningioma criteria (18) and interpretation by the local investigator.

This study will use the international Common Terminology Criteria for Adverse Events, version 5.0 (National Cancer Institute), for adverse event reporting.

QoL will be assessed using EORTC QoL questionnaires, cancer version 3 (QLQ-C30) and brain cancer (QLQ-BN20), as well as an EORTC item list (IL46) (19,20). Neurologic function will be assessed using the Neurologic Assessment in Neuro-Oncology scale (21). Frailty will be assessed with the Geriatric-8 score (French National Cancer Institute) in all patients age 70 y and older at baseline (22,23).

Tables 2–4 summarize the study calendar before treatment start, during treatment, and during follow-up, respectively. Data will be captured in electronic case report forms.

**TABLE 2.** Study Calendar Before Randomization

Task	Within 4 wk before enrollment	Within 2 wk before enrollment	Within 72 h before enrollment and within 7 d before treatment start
Informed consent	Mandatory		
Clinical examination*	Mandatory		
Medical history, demographics	Mandatory		
G8 assessment tool for patients ≥ 70 y of age	Mandatory		
Concomitant medication	Mandatory		
Electrocardiogram	Mandatory		
LVEF	Mandatory		
Hematology	Mandatory		
Serum chemistry	Mandatory		
Pregnancy test			Mandatory
Tumor evaluation using cranial MRI		Mandatory	
CT thorax or abdomen		If clinically relevant*	
Recording of corticosteroid dose	Mandatory		
NANO scale	Mandatory		
QLQ-C30, QLQ-BN20, and IL46	Mandatory		
SSTR PET	Mandatory		
FFPE collection for TR	Optional		

* In case of suspicion of or confirmed extracranial disease/metastases.

G8 = Geriatric-8; LVEF = left ventricular ejection fraction; NANO = Neurologic Assessment in Neuro-Oncology; FFPE = formalin-fixed paraffin-embedded tissue; TR = translational research.

**TABLE 3.** Study Calendar During Protocol Treatment

Task	Day 1 of each cycle or within 72 h before	At 24, 48, and 72 h after each infusion, experimental arm only	At end of second and fourth cycles after treatment initiation	Every 6 ± 1 wk from enrollment to follow-up
Clinical examination	Mandatory			Mandatory
Adverse events assessment	Mandatory			Mandatory
Concomitant medication	Continuous assessment			
Hematology	Mandatory			
Serum chemistry	Mandatory			
Electrocardiogram, LVEF	If clinically indicated			
Pregnancy test	Mandatory			
Disease evaluation				
Tumor evaluation using cranial MRI				Mandatory
CT thorax or abdomen				If clinically relevant
Recording of corticosteroid dose				Mandatory
Neurologic and QoL evaluation				
NANO scale				Mandatory
QLQ-C30, QLQ-BN20, and IL46				Mandatory
Optional investigations				
SSTR PET			Optional	
SPECT		Optional		

LVEF = left ventricular ejection fraction; NANO = Neurologic Assessment in Neuro-Oncology.

**TABLE 4.** Study Calendar at Follow-up

	In absence of progression			After disease progression
Task	Every 6 ± 1 wk until week 24, every 12 ± 2 wk thereafter up to 2 y or until week 96	Every 6 ± 1 wk during first 9 mo or until week 36, every 12 ± 2 wk thereafter	On suspicion of progression	Every 12 ± 2 wk
Survival		Mandatory		Mandatory
Clinical examination		Mandatory		
Adverse events assessment		Mandatory		Mandatory
Concomitant medication		Mandatory		Mandatory
Hematology		Mandatory		
Serum chemistry		Mandatory		
Pregnancy test	Mandatory			
Tumor evaluation using cranial MRI		Mandatory		
CT thorax or abdomen		If clinically relevant		
Recording of corticosteroid dose		Mandatory		
NANO scale	Mandatory			Mandatory
QLQ-C30, QLQ-BN20, and IL46	Mandatory			Mandatory
SSTR PET			Mandatory	
FFPE collection for TR				Optional

NANO = Neurologic Assessment in Neuro-Oncology; FFPE = formalin-fixed paraffin-embedded tissue; TR = translational research.

### Statistical Considerations

This trial is a randomized, comparative, phase II study with a Korn superiority design (24), comparing PFS between [^177^Lu]Lu-DOTATATE and local standard of care, with a treatment allocation ratio equal to 2:1 at randomization. Based on a Response Assessment in Neuro-Oncology review providing historical benchmarks for interventional therapy trials in surgery and radiation-refractory meningioma (25), it is assumed that 43% of patients will be grade 1 and 57% of patients will be grade 2 or 3. PFS at 6 mo equal to 25% is assumed in the local standard–of–care arm (control), and PFS at 6 mo equal to 46.7% is assumed in the [^177^Lu]Lu-DOTATATE group (i.e., a 21.7% difference). The primary endpoints will be analyzed on the basis of a direct comparison of PFS distribution based on the log-rank test and the hazard ratio obtained from the Cox model. PFS at 6 mo was used as the benchmark for statistical design but will not be formally compared and instead will be described only with the CI per arm. Assuming PFS follows an exponential distribution, this corresponds to a treatment hazard ratio equal to 0.55. On the basis of the log-rank test, a type I error equal to 10% 1-sided (20% 2-sided), and a power equal to 90% and assuming that 10% of patients will be excluded from the per-protocol population, the total sample size is 136 patients with histologically confirmed diagnosis of meningioma (all grades, grades 1–3 per WHO) and 99 PFS events need to be extracted from 122 patients in the per-protocol population. Stratification factors include country, WHO grade (grade 1 vs. grade 2 or 3), age (≤60 or >60 y), and tumor size (≤43 or >43 mm). The primary analysis will be performed in the intent-to-treat population, i.e., all randomized patients according to the arm to which they were allocated. In the per-protocol population, all randomized patients who have started their allocated treatment (≥1 dose of [^177^Lu]Lu-DOTATATE or the local standard–of–care drug or no treatment as part of the local standard of care) will be analyzed. In the per-protocol population, patients will be classified and analyzed in the arm they were allocated to by randomization. One interim analysis for futility (hazard ratio > 1) is planned when 33 PFS events are observed. At that time, it is expected that 70 patients are recruited, with 63 patients in the per-protocol population. The interim analysis should take place 18.7 mo from the first patient, encompassing the 15.7 mo needed to observe the required number of PFS events plus 3 mo to clean the database. There will be no accrual interruption for this analysis. As sensitivity analyses, all efficacy analyses, including PFS, will be repeated in the intent-to-treat population at a two-sided 5% significance, and PFS will be presented with an 80% CI.

### Interventions

Patients will be randomized by EORTC in a 2:1 ratio to the experimental arm of [^177^Lu]Lu-DOTATATE or to the control arm of local standard of care. In case of treatment discontinuation for reasons other than disease progression as part of this study, no further treatment is recommended before disease progression.

#### Experimental Arm: [^177^Lu]Lu-DOTATATE

[^177^Lu]Lu-DOTATATE will be intravenously administered at a dose of 7.4 GBq every 4 ± 1 wk for up to 4 cycles. The first infusion should be administered within 3 wk from enrollment. Standard guidelines for application of SSTR-targeted radionuclide therapy should be followed (26).

#### Control Arm: Local Standard of Care

Care in the control arm is left to the choice of the investigator. If a treatment is given, it should be initiated within 3 wk from enrollment. The following are the options to be used in the standard–of–care arm: hydroxycarbamide, bevacizumab, sunitinib, octreotide (Sandostatin LAR; Novartis), everolimus, or no antitumoral-specific treatment (observation and best supportive care).

Treatment in the local standard–of–care arm should be until progression or a maximum of 2 y from initiation of treatment, whichever comes first. Patients still benefiting from treatment, according to the treating physician, after 2 y may continue receiving treatment outside the study.

#### Supportive Care and Concomitant Medications

For renal protection purposes, a 2.5% lysine–arginine solution must be administered over 4 h concomitant to the [^177^Lu]Lu-DOTATATE infusion. Before the infusion with the 2.5% lysine–arginine solution is started, an intravenous or oral antiemetic is given according to local practice (e.g., granisetron, ondansetron, or tropisetron). Corticosteroids should be avoided as a preventive antiemetic treatment because of potential SSTR downregulation. In the local standard–of–care arm, monitoring and toxicity management guidelines applied locally should be followed.

### Translational Research

The trial protocol includes a comprehensive translational research program with dosimetry and imaging-based and tissue-based investigations with the following exploratory endpoints: tissue-based tumor characteristics (methylation, gene mutation profiling, and gene expression profiling); prognostic and predictive effect of SSTR PET uptake intensity parameters with outcome; correlation measured between instances of SSTR PET uptake; dosimetry parameters and outcome; change from baseline in health-related QoL in terms of role functioning, physical functioning, diarrhea, nausea, and vomiting; motor dysfunction and communication deficit at week 24; time to QoL deterioration for the 4 selected QoL scales of interest (global health QoL, cognitive functioning, social functioning, and fatigue) from EORTC QLQ-C30; change from baseline in health-related QoL in terms of all scales from EORTC QLQ-C30, QLQ-BN20, and IL46 at each assessment time point; validation of PET-based response criteria (Response Assessment in Neuro-Oncology) by assessing the correlation to MRI-based responses and as patient outcomes, including PFS and OS times; validation of MRI-based 3-dimensional growth rate models by assessing the correlation to PFS, OS, and patient clinical benefit; and correlation of tumor cell proliferation, microvascular density, and SSTR expression in relation to imaging features (MRI and PET) and treatment response (response rate, PFS, and OS) using immunohistochemistry. The imaging-based translational research program defined in the clinical trial protocol includes analyses on the prediction of tumor DNA methylation classes and molecular mutations of meningiomas based on MRI-derived radiomics features. Consent for collection and use of participant data and biologic specimens in these ancillary studies will be collected from each patient by the delegated investigator.

### Data Management

Data management principles of EORTC apply and have been described for other trial protocols (27). EORTC, in its role as sponsor and data controller of the clinical study, ensures that the processing activities on the personal data in the scope of this study are compliant with, but not limited to, the requirements set by a European Union General Data Protection Regulation (GDPR EU 2016/679), its subsequent amendments, and any additional national laws, recommendations, and guidelines.

This article does not contain individual personal data from patients. No identifying images or other personal or clinical details of participants are presented here or will be presented in reports of the trial results. The participant information materials and informed-consent form are available from the authors on request and after relevant contractual arrangements.

All data are collected via an electronic case report form by study staff with confirmed good clinical practice certification named on delegation logs at the trial sites and are stored in a secure database at EORTC Headquarters. The name of the patient will neither be asked for nor recorded at EORTC Headquarters. A sequential identification number will be automatically allocated to each patient registered in the trial. This number will identify the patient and will be included in all case report forms and corresponding material and data associated with the patient. To avoid identification errors, the patient’s code (maximum of 4 alphanumeric characters) and year of birth will also be reported on the case report forms. Data collected during the course of the research will be kept strictly confidential and accessed only by members of the trial team (or individuals from the sponsor organization or center sites where relevant to the trial).

### Study Governance

EORTC is the legal sponsor of the LUMEN-1 trial. The trial is performed as a research collaboration with Novartis, a pharmaceutical company, including the supply of financial support and the investigational drug by Novartis. EORTC has authority over the study design; collection, management, analysis, and interpretation of the data; writing of the report; and the decision to submit the report for publication, with contribution from the funder.

Study management principles of EORTC apply and have been described for other trial protocols (27). The Study Management Group consists of the EORTC Headquarters Team in charge of running the study and the principal study coordinators. The EORTC Headquarters Team is responsible for the day-to-day conduct of the trial. The Study Coordinator will assist the team in case of problems with patient evaluation (eligibility, treatment compliance, and safety). The Study Management Group also performs the medical review. If at any time during the course of the study the medical review identifies safety signals or other elements that could affect the potential risks and benefits to the study participants, these will be reported to the Study Steering Committee and may trigger a review by the EORTC Independent Data Monitoring Committee. The monitoring committee is in charge of the independent oversight of the trial and reports its recommendations in writing to the Study Management Group.

The Study Steering Committee for this study is composed of the study coordinators, further experts, and representatives of EORTC Headquarters (clinical scientist or statistician). This committee provides the general oversight of the study and has executive power. It monitors study progress and conduct. This committee will consider and act, as appropriate, on the recommendations of the Independent Data Monitoring Committee.

### Study Sites, Trial Status, and Timelines

Protocol version 1.0 was approved on April 29, 2024, and notification of all amendments will be given to the sites and to the competent authorities. LUMEN-1 was activated in February 2025. To achieve its accrual goal, the trial will enroll patients in 35 sites in 10 countries across Europe: Austria, Denmark, France, Germany, Italy, the Netherlands, Norway, Spain, Switzerland, and the United Kingdom (a list of study sites can be found at https://www.eortc.org/research_field/clinical-detail/2334/). The last patient enrollment is planned for the second quarter of 2027, the primary endpoint collection is planned for the fourth quarter of 2027, the secondary endpoint collection is planned for the third quarter of 2029, and the end-of-trial declaration is planned for the fourth quarter of 2029.

### Availability of Data and Materials

The datasets used or analyzed during the study will be made available according to EORTC policy. Plans for investigators and the sponsor to communicate trial results to participants, health care professionals, the public, and other relevant groups include presentations at scientific meetings, publications in peer-reviewed scientific journals, and information available to the public via the homepage, a newsletter, and social media postings of EORTC (https://www.eortc.org). EORTC is committed to ensuring that the data generated from its studies are put to good use by the cancer research community and, whenever possible, are translated to deliver patient benefit. Therefore, it is EORTC’s policy to consider sharing on request from qualified scientific and medical researchers all data generated from its research while safeguarding intellectual property, the privacy of patients, and confidentiality. Requests to access the data of published trials should be filed through the data-sharing tab on the EORTC website.

## DISCUSSION

To our knowledge, LUMEN-1 is the first prospective randomized trial evaluating a theranostic agent in neurooncology. As described in a recent position paper, the EORTC Brain Tumor Group has prioritized the development of this clinical trial because of the high unmet clinical need to find novel treatments for patients with recurrent meningioma, a highly expressed molecular target (i.e., SSTR2) with proven efficacy of a theranostic treatment ([^177^Lu]Lu-DOTATATE) in another tumor type (neuroendocrine tumors), and the lack of a relevant blood–brain or blood–tumor barrier (26). The objective of the LUMEN-1 phase II trial is to assess whether treatment with [^177^Lu]Lu-DOTATATE of patients with recurrent meningioma shows sufficient antitumor activity compared with the local standard of care to justify further investigation and development for routine clinical use in this indication.

The inclusion criteria of LUMEN-1 have been carefully defined to facilitate enrollment of a broad patient population that reflects routine practice in neurooncology and to maximize clinical relevance of the trial results. The study design of LUMEN-1 is based on the prior EORTC-BTG-1320 trial (NCT02234050), which was the first randomized trial enrolling patients with recurrent grade 2 or 3 meningiomas (28). Despite the relatively rare patient population and the challenging investigational agent (trabectedin chemotherapy administered intravenously over 24 h every 3 wk via a central venous catheter), the EORTC-BTG-1320 trial successfully completed accrual of 90 patients within 22 mo within a network of 35 sites from 9 countries in Europe. The LUMEN-1 trial will enroll not only patients with WHO grade 2 and 3 meningiomas but also patients with WHO grade 1 meningiomas, thus enabling screening of potential study participants from a larger patient pool. To facilitate adequate accrual of the overall target population of 136 patients, LUMEN-1 has been activated in 35 sites in 10 European countries.

Theranostic treatments enable selection of patients whose tumors show molecular target expression for goal-directed precision medicine therapy. For patient inclusion in LUMEN-1, evidence of SSTR expression is required on baseline PET scans. As an eligibility criterion, a pragmatic absolute SUV_max_ of more than 2.3, rather than other methods using the relative SUV, to compare liver or blood pool uptake was defined. At present, no consensus exists on the optimal methodology for the definition of SSTR PET tracer uptake for patient selection for radionuclide therapy (26). The threshold chosen for LUMEN-1 has been reported as the optimal threshold for differentiation between meningioma tissue and nonneoplastic tissue in a study systematically comparing uptake values on [^68^Ga]Ga-DOTATATE PET with histologic results based on neuronavigated tissue sampling (29). As an exploratory investigation, the prognostic and predictive effect of SSTR PET uptake intensity parameters with outcomes will be analyzed in the LUMEN-1 dataset.

As in EORTC-BTG-1320 (28), LUMEN-1 selects patients whose meningiomas have documented tumor growth in the 2 y before enrollment. This inclusion criterion is intended to ensure a homogeneous patient cohort with clearly progressive tumors in need of treatment. Most prior studies on meningiomas did not use such definitions at enrollment and thus were potentially biased by inclusion of patients with stable or only slowly growing tumors. Patients considered for LUMEN-1 enrollment must have had at least 1 prior neurosurgery and 1 line of external beam radiotherapy, but prior systemic therapy is not allowed. Thus, patients with early progression events, who potentially have tumors with relatively low secondary resistance, may be enrolled.

LUMEN-1 includes a comprehensive translational research program that will take advantage of clinical data, radiologic and PET imaging data, and tumor tissue samples collected in the frame of this prospective trial. To this end, a range of methods, such as immunohistochemistry, genetic and epigenetic tumor profiling, dosimetry, radiomics, and machine learning, will be used.

## CONCLUSION

LUMEN-1 will for the first time, to our knowledge, provide controlled data from a prospective randomized clinical trial on the efficacy of [^177^Lu]Lu-DOTATATE in patients with meningiomas recurring after neurosurgery and radiotherapy. It may open the path to novel treatment opportunities in this patient population.

## DISCLOSURE

The trial is performed with financial support from Novartis (https://www.novartis.com), a pharmaceutical company, including the investigational drug supplied by Novartis. Nathalie Albert has received honoraria for lectures, consultation, or advisory board participation from ABX, Advanced Accelerator Applications, Medsir, Novartis, OncLive, Servier, and Telix Pharmaceuticals and research funding from Novocure and Telix Pharmaceuticals. Emilie Le Rhun has received research grants from Bristol Myers Squibb and honoraria for lectures, advisory board participation, or consulting from AstraZeneca Daiichi, Bayer, Biodexa/Sitoxi, Janssen, Leo Pharma, Pfizer, Pierre Fabre, Roche, Seattle Genetics, and Servier. Osvaldo Mirante is an employee of Novartis or owns Novartis stocks or shares. Michael Weller has received research grants from Novartis, Quercis, and Versameb and honoraria for lectures, advisory board participation, or consulting from AnHeart, Bayer, CureVac, Medac, Neurosense, Novartis, Novocure, Orbus, Pfizer, Philogen, Roche, and Servier. Joerg-Christian Tonn has received travel grants from Servier and honoraria for consultation from Novartis. Matthias Preusser has received honoraria for lectures, consultation, or advisory board participation from the following for-profit companies: Bayer, Bristol Myers Squibb, Novartis, Gerson Lehrman Group, CMC Contrast, GlaxoSmithKline, Mundipharma, Roche, BMJ Journals, MedMedia, AstraZeneca, AbbVie, Lilly, MedAhead, Daiichi Sankyo, Sanofi, Merck Sharp & Dohme, Tocagen, Adastra, Gan & Lee Pharmaceuticals, Janssen, Servier, Miltenyi, Boehringer Ingelheim, Telix, Medscape, and OncLive. No other potential conflict of interest relevant to this article was reported.
